# The Camden & Islington Research Database: Using electronic mental health records for research

**DOI:** 10.1371/journal.pone.0190703

**Published:** 2018-01-29

**Authors:** Nomi Werbeloff, David P. J. Osborn, Rashmi Patel, Matthew Taylor, Robert Stewart, Matthew Broadbent, Joseph F. Hayes

**Affiliations:** 1 UCL Division of Psychiatry, University College London, London, United Kingdom; 2 Camden and Islington NHS Foundation Trust, London, United Kingdom; 3 Institute of Psychiatry, Psychology and Neuroscience, King’s College London, London, United Kingdom; 4 South London and Maudsley NHS Foundation Trust, London, United Kingdom; Department of Psychiatry and Neuropsychology, Maastricht University Medical Center, NETHERLANDS

## Abstract

**Background:**

Electronic health records (EHRs) are widely used in mental health services. Case registers using EHRs from secondary mental healthcare have the potential to deliver large-scale projects evaluating mental health outcomes in real-world clinical populations.

**Methods:**

We describe the Camden and Islington NHS Foundation Trust (C&I) Research Database which uses the Clinical Record Interactive Search (CRIS) tool to extract and de-identify routinely collected clinical information from a large UK provider of secondary mental healthcare, and demonstrate its capabilities to answer a clinical research question regarding time to diagnosis and treatment of bipolar disorder.

**Results:**

The C&I Research Database contains records from 108,168 mental health patients, of which 23,538 were receiving active care. The characteristics of the patient population are compared to those of the catchment area, of London, and of England as a whole. The median time to diagnosis of bipolar disorder was 76 days (interquartile range: 17–391) and median time to treatment was 37 days (interquartile range: 5–194). Compulsory admission under the UK Mental Health Act was associated with shorter intervals to diagnosis and treatment. Prior diagnoses of other psychiatric disorders were associated with longer intervals to diagnosis, though prior diagnoses of schizophrenia and related disorders were associated with decreased time to treatment.

**Conclusions:**

The CRIS tool, developed by the South London and Maudsley NHS Foundation Trust (SLaM) Biomedical Research Centre (BRC), functioned very well at C&I. It is reassuring that data from different organizations deliver similar results, and that applications developed in one Trust can then be successfully deployed in another. The information can be retrieved in a quicker and more efficient fashion than more traditional methods of health research. The findings support the secondary use of EHRs for large-scale mental health research in naturalistic samples and settings investigated across large, diverse geographical areas.

## Introduction

Case registers have been an important component of mental health research since its origins in the late 19th century. The extensive data contained in psychiatric case registers can be used for the development of public health care policy, quality control, epidemiological and service research [1]. Case registers are particularly helpful when investigating the course and outcome of a disorder, as well as allowing intervention response to be evaluated in large, naturalistic samples and settings [2].

Electronic health records (EHRs) have become widely used in UK mental health clinical practice and, therefore, new sources of detailed clinical information are available [3]. EHRs are composed of clinical information captured electronically as structured data as well as data within “free text”. The secondary use of data collected in EHRs can dramatically increase the breadth and depth of information available for research [3, 4]. A case register using EHRs from secondary mental healthcare as source data has the potential to deliver large-scale projects evaluating mental health outcomes in real-world clinical populations, with long follow-up periods. Such studies would otherwise be unfeasible to conduct in terms of time, resource and funding.

A mental health case register was developed and implemented in 2008 across South London and Maudsley NHS Foundation Trust (SLaM), a large secondary mental healthcare provider [5]. The Clinical Record Interactive Search (CRIS) is a tool developed by SLAM Biomedical Research Centre (BRC) to enable routinely collected EHRs to be used in research, using an explicit de-identification process [6]. In 2013, funded by National Institute for Health Research (NIHR) D-CRIS programme, the CRIS tool was deployed to four additional Trusts in the UK, including Camden and Islington NHS Foundation Trust (C&I). This paper describes the implementation of CRIS outside SLaM, and tests the feasibility of using a tool developed by one mental healthcare organization in a different mental healthcare setting. The paper describes the C&I Research Database and demonstrates its functioning and capabilities by assessing whether a research project originally conducted on the SLaM case register can be repeated with the same degree of success in a different data source, namely the C&I Research Database. The research project examines time to diagnosis and treatment of bipolar disorder, using applications for identification of diagnoses and medication in free text records.

## Materials and methods

### Camden and Islington NHS Foundation Trust

C&I is a large mental healthcare provider serving a geographic catchment area of two inner-city London boroughs, and approximately 470,000 residents. Based on social deprivation scores of 326 local authorities in England, Camden is the 74th and Islington is the 14th most deprived local authority [7]. The variation in the levels of deprivation within both boroughs is large, highlighting the inequalities between different population groups and places [8]. Within Camden there are areas that are within the top 10% most deprived areas in England and areas that are in the 20% least deprived [9].

Descriptive data for the two boroughs are summarised in Table 1 and compared with those for London and England. The C&I catchment boroughs do not differ substantially from greater London in terms of the sex distribution, though the proportion of younger people (under the age of 35) is higher in the two boroughs as compared with both London and England as a whole. Overall, in greater London, 59.8% of the population are White and 40.2% are Black, Asian, and minority ethnic (substantially higher compared to England). The proportion of white people is slightly higher in the two boroughs included in this study: Camden—66.3% and Islington—68.0%.

**Table 1:** Descriptive statistics for Camden & Islington, compared with statistics for London and England as a whole.

		C&I catchment		Comparison statistics	
		Camden	Islington	London	England
Total population*		241,059	227,692	8,673,713	54,786,327
Sex*	M	50.0%	50.0%	49.7%	49.3%
	F	50.0%	50.0%	50.3%	50.7%
Age*	15–24	14.4%	15.4%	12.0%	12.4%
	25–34	22.9%	27.9%	20.4%	14.3%
	35–44	15.7%	15.2%	15.7%	13.0%
	45–54	11.7%	11.6%	12.9%	14.1%
	55–64	8.3%	7.3%	8.9%	11.3%
	65–74	6.5%	4.9%	6.2%	9.6%
	75–84	3.6%	2.8%	3.8%	5.7%
	85+	1.6%	1.0%	1.6%	2.4%
Ethnicity #	White	66.3%	68.0%	59.8%	85.5%
	Asian / Asian British	16.1%	9.0%	18.4%	7.7%
	Black / Black British	8.2%	13.0%	13.3%	3.4%
	Mixed	5.6%	6.0%	5.1%	2.2%
	Other	3.5%	4.0%	3.4%	1.2%

* ONS 2014 mid-year population estimates (Data source: https://data.london.gov.uk/dataset/ons-mid-year-population-estimates-custom-age-tables)

^#^ ONS 2011 census data

C&I provide mental health and substance misuse services to people living in Camden and Islington, substance misuse services to Westminster, and a substance misuse and psychological therapies service to residents in Kingston. The Trust has two inpatient facilities, at Highgate Mental Health Centre and St Pancras Hospital, as well as community based services throughout the London boroughs of Camden and Islington. The Trust provides services for adults of working age, adults with learning difficulties, and older people in community or inpatient settings.

### The C&I Research Database

Routine recording of EHRs at C&I had commenced in mid-2008 using RiO, an electronic patient record system. RiO contains a comprehensive, longitudinal record of all clinical information recorded throughout patients’ contacts with Trust services, including socio-demographic information, dates and other details of referrals and admissions, detailed clinical assessments, care plans and standardized assessment forms (such as the Risk Assessment [10] and Health of The Nation Outcome Scales [11]). The record consists of both structured fields (such as dates and pick-lists) and unstructured free text (including progress notes and correspondence).

The CRIS tool, developed by SLAM BRC to extract information from their bespoke electronic Patient Journey System (PJS), consists of a series of data-processing pipelines which both structure and de-identify fields in the EHR, rendering effectively anonymized data from the full clinical record available at the researcher interface (2). The system allows researchers to search against any combination of structured and unstructured fields that exists in the database. Users then specify the precise fields they want returned (such as specific diagnostic codes, demographic information and/or a particular text string in a clinical assessment) (5).

The C&I Research Database employs the same security model as that developed by SLaM to address the legal and ethical considerations attendant upon the use of confidential health data [5]. Authorized researchers are provided with regulated access to anonymized information extracted from patient EHRs. The research database is used to support epidemiological and population-based research using only anonymized data, for which no patient consent is necessary though patients can opt out entirely if they choose. Studies using the C&I Research Database received ethical approval from the NRES Committee East of England—Cambridge Central (14/EE/0177).

Access to the database is overseen by an Oversight Committee including stakeholders and patient leadership. The Committee oversees approvals for researchers and studies; ensures that queries do not carry a significant potential for de-anonymization; and monitors use of the database to ensure that studies are conducted in accordance with information governance regulations. The database administrator works closely with the Oversight Committee and the Trust’s R&D division on the day to day management of the Research Database.

### Data extraction from free text

Natural language processing (NLP) techniques have been developed and applied by SLAM BRC, in collaboration with University of Sheffield Department of Computer Science, for extracting knowledge from unstructured text captured in clinical notes and correspondence. The development of these techniques has been described elsewhere [2]. Several NLP applications using rule based pattern matching of key concepts have been applied to the C&I Research Database. These applications were built using General Architecture for Text Engineering (GATE), a widely used program which provides a suite of tools to assist with NLP tasks such as information extraction from clinical notes. These applications were designed to extract data from the free text taking into account the linguistic context of a word or phrase of interest, thus allowing structured data to be obtained from free text fields.

### Analysis plan

#### Cohort profile

Descriptive statistics for the sex, age and ethnicity of all patients included in the C&I Research Database on the 31^st^ of August 2015 were examined. Patients receiving active care were defined as those who had joined the Trust in the previous year or who had open referrals, admissions or care-plans recorded in the preceding year. The demographic characteristics of patients receiving active care were examined separately.

#### Time to diagnosis and initiation of treatment in patients presenting to mental health services with bipolar disorder

To assess the capabilities of the C&I Research Database and cross-site comparability we sought to replicate a study conducted by Patel et al. [12] examining time to diagnosis and treatment of bipolar disorder using the SLAM BRC case register. For this purpose, we identified a cohort of individuals meeting the following criteria from the C&I Research Database:

1. First presentation to C&I between 1st January 2009 and 31st August 2014.
2. Age between 16 and 65 years at first presentation.
3. Subsequent diagnosis of mania or bipolar affective disorder before 31st August 2015.

Diagnoses of mania and bipolar disorder were derived from EHR structured fields containing ICD-10 diagnoses (F30.x and F31.x). This information was then supplemented with diagnostic data retrieved from free text fields using NLP. The NLP application for ‘diagnosis’ extracts text strings associated with a diagnosis statement in order to supplement the existing structured fields. We tested the positive predictive value (PPV) of the application for extracting and coding diagnosis data on randomly selected instances where the application coded the patient as being diagnosed with bipolar disorder (*n* = 100). We then determined if this was correct by manually searching through the underlying document. To determine sensitivity we extracted a random set of documents (*n* = 100) that contained the words ‘bipolar disorder’, read these documents to ascertain whether the patient was actually diagnosed with bipolar disorder, then determined if this was in agreement with the coding performed by the NLP application.

In order to ensure that participants included in the analysis had a stable diagnosis of bipolar disorder, only participants whose mania or bipolar disorder diagnosis was confirmed at least once within one year of initial diagnosis were included. The time period of 2009 to August 2014 was chosen as 2009 was the first full year in which EHRs were implemented in C&I, and to ensure that all individuals in the study had at least one year of follow-up data available.

The CRIS tool was used to extract the following predictor variables from the dataset: 1) whether the patient was admitted to hospital compulsorily under the UK Mental Health Act (MHA) within 2 weeks of first presentation to C&I, and 2) whether the patient received a diagnosis of schizophrenia or related disorders (ICD-10 F2x), psychotic depression (ICD-10 F32.3/F33.3), unipolar depression without psychotic symptoms (ICD-10 F32/33 excluding F3x.3), anxiety disorder (ICD-10 F4x), personality disorder (ICD-10 F60/F61), alcohol or illicit drug misuse/dependence (ICD-10 F10.x, F11-19.x) prior to the date of first bipolar disorder diagnosis.

The following variables were extracted as covariates for multivariable analyses: age, sex, ethnicity and marital status. All covariate data obtained were those closest to the time of first referral to C&I.

The primary outcome variable was time to diagnosis of bipolar disorder (in days) measured from the date of first presentation to C&I. We considered this time to represent the interval to diagnosis of bipolar disorder while receiving specialist mental healthcare. The secondary outcome variable was time to first prescription of appropriate treatment (in days) measured from the date of first presentation to C&I. We considered this time to represent the interval to initiating treatment. As defined by Patel et al. [12], and in reference to the British Association of Psychopharmacology guidelines [13], appropriate pharmacological treatment included initiation of any of second generation antipsychotic, lithium, valproate, carbamazepine and lamotrigine identified using the NLP application for ‘medication’.

Associations between predictor variables and time to diagnosis and treatment were investigated using Kaplan-Meier survival analysis and multivariable Cox regression. The hazard ratios in Cox regression analyses represent the probability of bipolar disorder diagnosis or initiation of treatment occurring during the period of follow-up. Therefore, a hazard ratio greater than 1.0 indicates an association of a predictor variable with reduced time to diagnosis or treatment compared to the reference category. Reference categories for Cox regression analysis were defined as those of greatest prevalence. By virtue of the study inclusion criteria, all participants were diagnosed with bipolar disorder and so no censoring was required in the survival analysis of time to diagnosis (time to first record of bipolar disorder in structured field or free text). For analysis of time to treatment, the outcome of starting appropriate treatment was censored at 31st August 2015. Data were analyzed using SPSS version 22.0 [14].

## Results

### Cohort profile

Table 2 shows summary statistics of C&I Research Database for the years 2008 -31st August 2015. The largest age group of patients was 35–44 years. There were slightly more females than males represented in the database. While the majority of patients were from white ethnic backgrounds, approximately a third of the patients did not have ethnicity recorded in their EHRs, making comparisons to the ethnic composition of the C&I boroughs difficult.

**Table 2:** Demographic characteristics of patients in the C&I Research Database.

		All Patients (n = 108,168)	Active patients^1^ (n = 23,538)
Sex	M	52,243 (48.4%)	11,726 (49.8%)
	F	55,718 (51.5%)	11,806 (50.2%)
	missing	159 (0.1%)	6 (0.02%)
Age	15–24	5,302 (4.9%)	2,258 (9.6%)
	25–34	19,854 (18.4%)	4,620 (19.6%)
	35–44	24,665 (22.8%)	4,587 (19.5%)
	45–54	22,295 (20.6%)	4,546 (19.3%)
	55–64	12,725 (11.8%)	2,553 (10.8%)
	65–74	7,838 (7.2%)	1,552 (6.6%)
	75–84	6,266 (5.8%)	1,842 (7.8%)
	85+	9,175 (8.5%)	1,580 (6.7%)
Ethnicity	White	51,713 (47.8%)	13,560 (57.7%)
	Asian / Asian British	4,136 (3.8%)	1,219 (5.2%)
	Black / Black British	7,620 (7.0%)	2,539 (10.8%)
	Mixed	1,923 (1.8%)	695 (3.0%)
	Other	5,737 (5.3%)	1,597 (6.8%)
	Missing	36,991 (34.2%)	3,928 (16.7%)

^1^ Patient receiving active care are those who had joined the Trust in the last year or who had open referrals, admissions or care-plans recorded in the last year

On the 31st August 2015, the number of patients receiving active care (defined as those who had joined the Trust in the last year or who had open referrals, admissions or care-plans recorded in the last year) was 23,538. Active patients were slightly younger than the entire cohort (with a higher proportion aged 15–34), and had fewer missing data.

### Time to diagnosis and initiation of treatment in patients presenting to mental health services with bipolar disorder

We identified a cohort of 467 individuals meeting the inclusion criteria for this study. A comparison of the study population identified at C&I and that identified at SLaM by Patel et al. [12] is presented in Table 3.

**Table 3:** Time to diagnosis and treatment of bipolar disorder—Comparison between C&I and SLaM cohorts.

	C&I	SLaM
Residents in catchment area	470,000	1,200,000
Period of first presentation to Trust	1st January 2009–31st August 2014	1st January 2007–31st December 2012
No. of patients with bipolar disorder meeting inclusion criteria	467 (0.10%)*	1364 (0.11%)
No. of patients with bipolar disorder receiving appropriate treatment by end of follow-up	395 (85%)#	1206 (88%)
Median time to diagnosis (IQR), days	76 (17–391)	62 (17–243)
Median time to treatment (IQR), days	37 (5–194)	31 (4–122)

* Percent of residents in the catchment area who meet inclusion criteria

^#^ Percent of patients with bipolar disorder who received appropriate treatment by the end of follow-up

#### Performance of NLP application for the detection of bipolar disorder

The NLP ‘diagnosis’ application was able to identify instances of bipolar disorder with high PPV, although sensitivity levels were more modest. The PPV obtained from the validation set of 100 patients was 0.92, and sensitivity was 0.64.

#### Time to diagnosis

**Fig 1:**
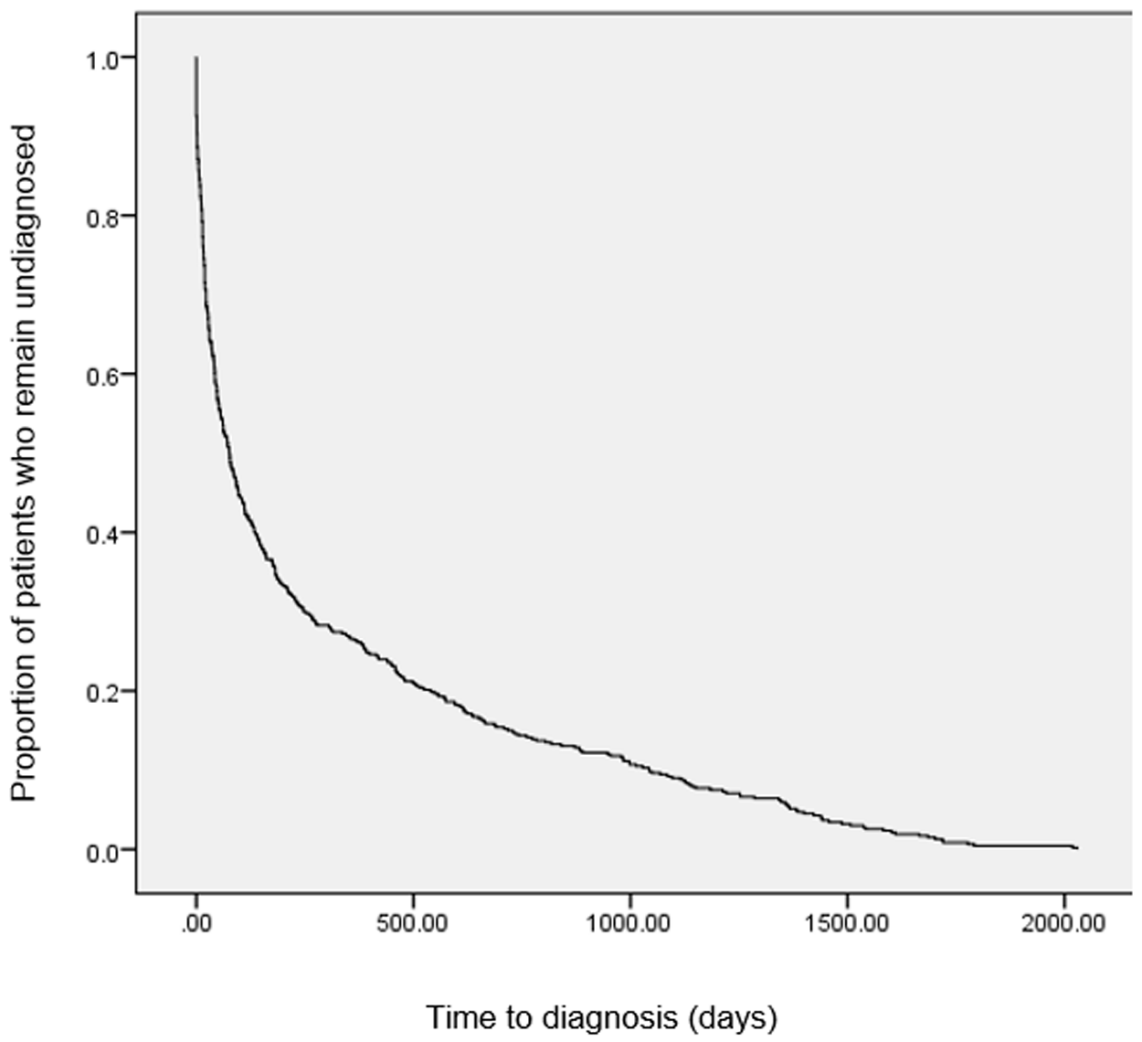
Kaplan-Meier plot of time to diagnosis.

The median interval to diagnosis of bipolar disorder was 76 days (IQR 17–391). Kaplan-Meier analysis illustrates the distribution of intervals to diagnosis over time (Fig 1). The interval identified at SLaM was slightly shorter (median = 62, IQR 17–243 days [12]; Table 3).

Table 4 shows the breakdown of time to diagnosis according to demographic and clinical characteristics. Table 5 shows the associations between patient characteristics and time to diagnosis. As in the SLaM cohort, there were no significant associations between time to diagnosis and age, sex, ethnicity or marital status. Patients with missing data on either ethnicity or marital status had shorter intervals to diagnosis as compared to their counterparts. Prior diagnoses of other psychiatric disorders were associated with longer intervals to diagnosis compared to people without these prior diagnoses. In particular, prior diagnoses of personality disorders were associated with substantially longer median intervals to diagnosis. Compulsory admission under the UK MHA was associated with shorter interval to diagnosis.

**Table 4:** Time to diagnosis and treatment of bipolar disorder by demographic and clinical characteristics.

Factor	Group	Number in sample	Percentage	Median Interval to diagnosis in days (IQR)	Median Interval to treatment in days (IQR)*
Age (years)	<25	122	26.1%	91 (20–519)	44 (8–203)
	26–35	168	36.0%	55 (14–302)	45 (5–221)
	36–45	108	23.1%	74 (14–340)	24 (4–188)
	46–55	47	10.1%	56 (27–336)	43 (4–138)
	56–65	22	4.7%	164 (50–331)	38 (8–216)
Sex	Female	276	59.1%	78 (17–414)	45 (6–244)
	Male	191	40.9%	76 (17–386)	34 (5–135)
Ethnicity	White	300	64.2%	87 (19–502)	47 (6–212)
	Asian	21	4.5%	43 (2–529)	26 (6–344)
	Black	36	7.7%	110 (27–298)	34 (8–246)
	Other	55	11.5%	28 (12–419)	16 (2–160)
	Not recorded	55	11.5%	40 (11–133)	35 (3–138)
Marital status	Married/Cohabiting	59	12.6%	141 (18–574)	43 (5–331)
	Divorced/Separated	38	8.1%	60 (30–211)	55 (18–137)
	Single	281	60.2%	97 (21–494)	44 (7–217)
	Widowed	2	0.4%	-	-
	Not recorded	87	18.6%	22 (2–85)	10 (1–112)
UK Mental Health Act	Compulsory admission within 2 weeks of first presentation	70	15.0%	11 (1–30)	3 (1–8)
Prior Diagnosis	Schizophrenia or related disorders	40	8.6%	414 (144–888)	12 (2–177)
	Psychotic depression	5	1.1%	547 (214–1136)	10 (3–68)
	Unipolar depression without psychotic symptoms	39	8.4%	449 (133–1028)	125 (24–287)
	Anxiety disorder	25	5.4%	503 (206–955)	48 (4–200)
	Personality disorder	18	3.9%	983 (137–1609)	175 (45–702)
	Alcohol / drug misuse or dependence	20	4.3%	224 (109–764)	102 (7–213)

*Includes only 395 patients who received appropriate treatment during the follow-up period

**Table 5:** Factors associated with time to diagnosis and treatment of bipolar disorder (n = 467)—Multivariable cox regression model.

Factor	Group	Interval to diagnosis		Interval to treatment	
		*Adjusted hazard ratio	95% confidence interval, p value	*Adjusted hazard ratio	95% confidence interval, p value
Age (years)	<25	0.89	0.70–1.14, p = 0.34	0.86	0.66–1.12, p = 0.27
	26–35	Reference		Reference	
	36–45	1.00	0.78–1.29, p = 0.996	0.94	0.72–1.23, p = 0.64
	46–55	0.90	0.64–1.27, p = 0.55	0.86	0.59–1.27, p = 0.45
	56–65	0.88	0.56–1.38, p = 0.57	1.30	0.80–2.11, p = 0.29
Sex	Female	Reference		Reference	
	Male	0.92	0.95–1.12, p = 0.39	0.89	0.72–1.11, p = 0.30
Ethnicity	White	Reference		Reference	
	Asian	1.32	0.84–2.08, p = 0.23	1.23	0.76–2.00, p = 0.41
	Black	0.98	0.68–1.41, p = 0.90	0.99	0.67–1.47, p = 0.95
	Other	1.11	0.82–1.50, p = 0.50	1.15	0.84–1.58, p = 0.39
	Not recorded	1.59	1.15–2.20, p = 0.005	1.07	0.75–1.52, p = 0.72
Marital status	Married/Cohabiting	0.85	0.63–1.16, p = 0.31	0.92	0.66–1.27, p = 0.60
	Divorced/Separated	1.11	0.77–1.58, p = 0.58	1.15	0.79–1.67, p = 0.48
	Single	Reference		Reference	
	Widowed	2.31	0.55–9.59, p = 0.25	2.02	0.48–8.46, p = 0.34
	Not recorded	1.58	1.20–2.08, p = 0.001	0.91	0.67–1.23, p = 0.53
UK Mental Health Act	Compulsory admission within 2 weeks of first presentation	3.92	2.90–5.30, p < .001	4.03	2.98–5.47, p < .001
Prior Diagnosis	Schizophrenia or related disorders	0.40	0.27–0.57, p < .001	1.52	1.05–2.20, P = 0.025
	Psychotic depression	0.80	0.32–2.00, p = 0.64	2.31	0.86–6.23, p = 0.097
	Unipolar depression without psychotic symptoms	0.64	0.45–0.91, p = 0.01	0.90	0.62–1.32, p = 0.59
	Anxiety disorder	0.59	0.39–0.91, p = 0.02	1.15	0.72–1.82, p = 0.56
	Personality disorder	0.41	0.24–0.68, p = 0.001	0.80	0.47–1.36, p = 0.42
	Alcohol / drug misuse or dependence	0.89	0.55–1.41, p = 0.59	1.12	0.67–1.89, p = 0.67

*Model adjusted for all factors listed in this table

#### Time to treatment

**Fig 2:**
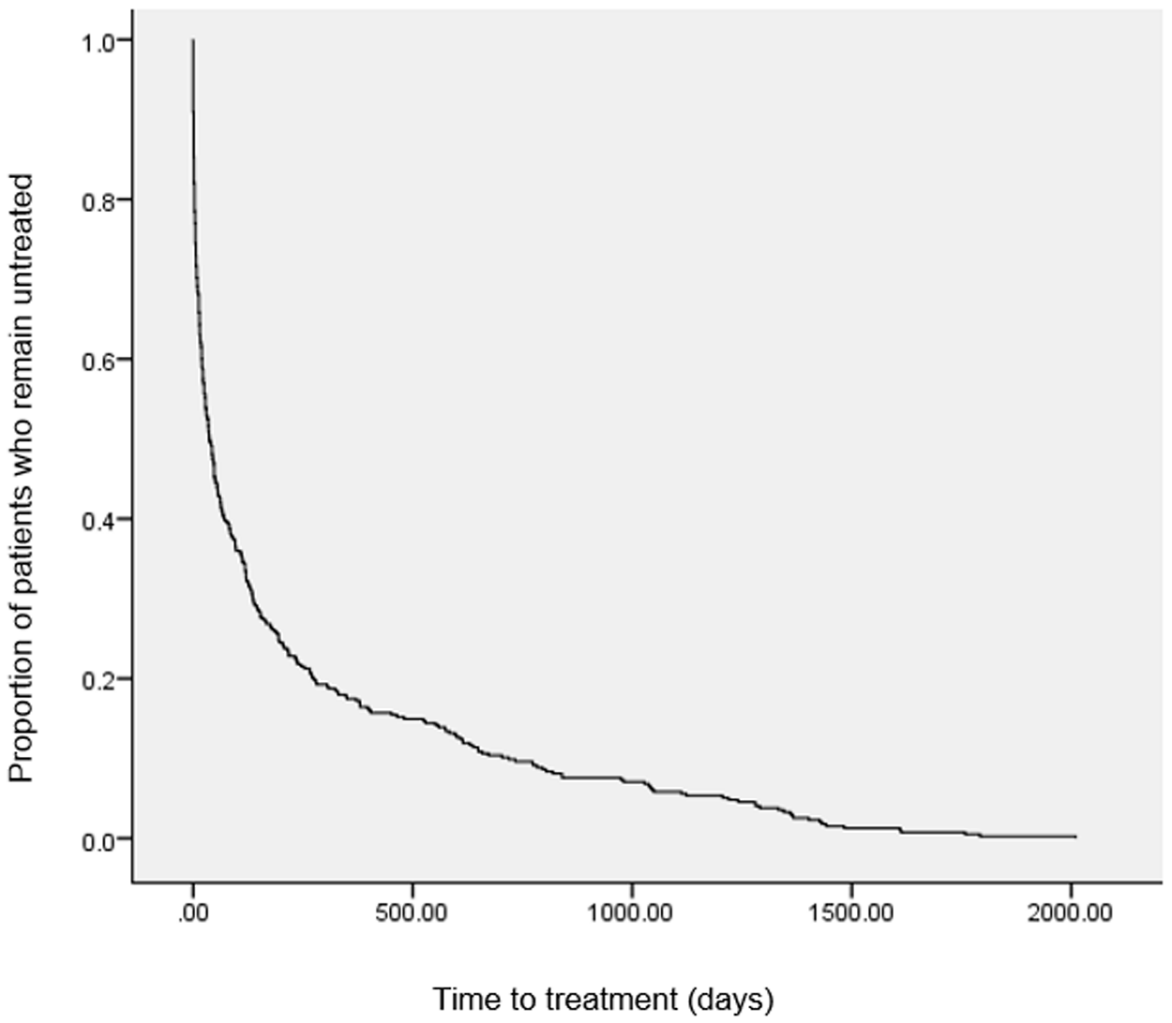
Kaplan-Meier plot of time to treatment.

Of the 467 individuals included in this study, 395 received appropriate treatment prior to 31st August 2015. The median interval to treatment was 37 days (IQR 5–194), comparable to that found at SLaM (median = 31, IQR 4–122 days [12]; Table 3). 196 individuals (54.6%) were found to have shorter time to treatment than time to diagnosis. Kaplan-Meier analysis illustrates the distribution of intervals to treatment over time (Fig 2).

Time to Treatment by demographic and clinical characteristics is presented in Table 4, and the associations between these characteristics and time to treatment is presented in Table 5. As in the SLaM cohort, there were no significant associations between time to treatment and age, sex, ethnicity or marital status. In contrast to time to diagnosis, prior diagnoses of schizophrenia and related disorders were significantly associated with a reduction in median interval to treatment. Compulsory admission under the UK MHA was associated with shorter interval to treatment.

## Discussion

This paper describes the properties of a psychiatric case register containing fully EHRs from a large UK secondary mental healthcare provider. The case register is an effective data source for studying patterns of care, providing invaluable information for evaluating/auditing and planning services, as well as monitoring service use [5]. The information can be retrieved in a much quicker and more efficient fashion than more traditional methods of record keeping.

Currently, the C&I Research Database contains over 108,000 patient records. The database is routinely updated, cleaned and anonymized, enabling users to search and extract anonymized data. The strengths of the database lie in its size, breadth, long-term follow-up, and representativeness of the population in the two boroughs (in the UK the vast majority of mental healthcare is provided by specific state-run services with minimal use of privately funded care). The limitations stem from the fact that these are routinely collected health records rather than data collected specifically for research purposes. Information incompleteness, inaccuracy, and inconsistency are challenges, and data quality is largely dependent on the healthcare professionals who record the clinical information.

Many patients accessing secondary mental healthcare will receive diagnosis and treatment in primary care. Additional information relating to patients’ morbidity and mortality may not be available within mental health EHRs but rather stored in external databases. Thus, future plans for the C&I Research Database include linkage with external data sources. SLaM’s experience with data linkages (2) suggests that it has the potential to enhance existing data and expand the depth of information available in mental health case registers. However, data linkage also poses several challenges including different regulatory processes across each of the data providers, mismatched identifier variables which may limit the linkage process, and the sheer volume of data to be processed.

Much of the clinical information in EHRs is recorded in free text fields. Free text is often richer in detail but requires specialized approaches, including NLP, to extract clinically relevant concepts [15]. Such techniques have been applied in the C&I Research Database enabling the retrieval of information from free text. In the context of this paper we evaluated the performance of an NLP application designed to extract diagnoses from free text fields. The diagnosis of bipolar disorder showed high PPV and moderate sensitivity. Employing text mining within this data set has involved a trade-off between PPV and sensitivity. However, the longitudinal nature of EHR data means that there are generally multiple opportunities for an NLP application to capture a piece of information; therefore, suboptimal sensitivity can be compensated for and the focus has been on maximising PPV [2].

Next, to illustrate the capabilities of the C&I Research Database, we investigated time to diagnosis and treatment of bipolar disorder from initiation of specialist mental healthcare. The median interval to diagnosis from the point of receiving specialist mental healthcare was slightly longer at C&I compared to SLaM [12] with a wider distribution, partially due to the smaller sample size. A similar trend was observed for the interval to receiving appropriate treatment for bipolar disorder. As suggested by Patel et al [12], the fact that time to treatment was shorter than time to diagnosis for the majority of patients may reflect the initiation of treatment by clinicians prior to recording a formal diagnosis of bipolar disorder in the EHR. It is important to note that it was not possible to determine whether first contact with specialist mental healthcare was based on presenting bipolar symptoms for the index diagnostic episode or for other/ pre-existing mental health problems. The distinction between these may have implications on time to diagnosis and treatment.

Similar to the results using the SLaM case register, we found no significant association of age, sex, ethnicity or marital status with time to diagnosis or treatment. Patients who underwent compulsory admission to hospital under the UK MHA had a shorter interval to diagnosis and treatment. Admission under the UK MHA may be a marker of illness severity, requiring prompt treatment and facilitating a more timely diagnosis.

Prior diagnoses of other psychiatric disorders were associated, for the most part, with longer time to diagnosis, particularly in the case of prior diagnoses of personality disorders. It is possible that early symptoms of bipolar disorder are often misattributed to other psychiatric conditions. This is in line with the finding of the National Depressive and Manic-Depressive Association reporting that many patients with bipolar disorder are misdiagnosed initially [16], and the evidence of high comorbidity of bipolar disorder with other psychiatric diagnoses [17].

Contrary to this, prior diagnoses of schizophrenia and related disorders were significantly associated with a reduction in median interval to treatment. This could be explained by the use of second generation antipsychotics to treat psychotic disorders (which are also indicated in the treatment of bipolar disorder); hence, the increased time to diagnosis is not reflected in a parallel increased time to treatment.

In summary, this paper aimed to describe the C&I Research Database and demonstrate its capabilities. While the C&I Research Database contains data from a single mental healthcare provider in a geographical catchment area, results are similar to those observed in SLaM—a mental healthcare provider covering a much larger catchment area with a diverse population. It is reassuring that data from different organizations deliver similar results, and that NLP applications developed in one Trust can then be successfully deployed in another. It is also encouraging that the CRIS tool which was developed to extract data from SLaM’s bespoke EHR system—PJS—worked equally well on a different EHR system, namely RiO. The findings support the secondary use of EHRs for large-scale research in mental health. Such data can enable new insights into mental health processes and outcomes investigated across large, diverse geographical areas, answering questions of regional, national and international importance.
